# The effect of general anesthetics on neutrophil-like differentiated HL60 cells: sevoflurane activates the mitochondrial function to promote their bactericidal action

**DOI:** 10.1016/j.bbrep.2025.102272

**Published:** 2025-09-19

**Authors:** Kosuke Asei, Yuki Nomura, Daichi Fujimoto, Mayu Ooi, Norihiko Obata, Satoshi Mizobuchi

**Affiliations:** Department of Anesthesiology, Kobe University Graduate School of Medicine, Kobe, Hyogo, Japan

**Keywords:** Neutrophil-like differentiated HL60 cell, General anesthetics, Mitochondrial metabolism, Mitochondrial respiration, Reactive oxygen species

## Abstract

**Background:**

Recent research has suggested that general anesthetics may affect the immune system, but it is unclear how they affect the mitochondria of neutrophils. The effects of general anesthetics on neutrophil-like differentiated HL60 cells mitochondrial function and their production of reactive oxygen species (ROS) were examined in this study.

**Methods:**

HL60 cells were differentiated into neutrophil-like cells by treating them with 1 μM all-trans-retinoic acid for 4 days. The differentiated HL60 cells were exposed to propofol (4 μg ml^−1^), midazolam (0.5 μg ml^−1^), or sevoflurane (3 %) for 1 h or 5 h. Following the exposure to the anesthetics, we analyzed the production of ROS, bactericidal effects, cell viability, and apoptosis of the neutrophil-like differentiated HL60 cells. We also investigated the effects of these anesthetics on mitochondrial morphology and function of the neutrophil-like differentiated HL60 cells.

**Results:**

Administration of propofol or midazolam activated neutrophil-like differentiated HL60 cells mitochondrial respiration and increased ATP production (P < 0.05). However, there was no significant change in production of ROS or cell death. Exposure to sevoflurane activated mitochondrial respiration, ATP production (P < 0.05) and ROS production (P < 0.05). Additionally, exposure to sevoflurane led to a significant increase in phagocytosis. Filamentous mitochondria appeared after treatment with propofol, midazolam, and sevoflurane, while fragmented and filamentous mitochondria were seen in untreated neutrophil-like differentiated HL60 cells.

**Conclusion:**

We investigated the effects of three anesthetics on the mitochondrial and bactericidal functions of differentiated HL60 cells. Our results indicate that sevoflurane activates mitochondrial function in differentiated HL60 cells, promotes the production of reactive oxygen species, and enhances bactericidal activity.

## Highlights

• We investigated the effects of three anesthetics on the mitochondrial and bactericidal functions of differentiated HL60 cells.
• Propofol and midazolam activate mitochondrial function in differentiated HL60 cells without significant changes in the production of reactive oxygen species.
• Sevoflurane activates mitochondrial function in differentiated HL60 cells, promotes the production of reactive oxygen species, and enhances bactericidal activity.

## Graphical abstract

**Figure:**
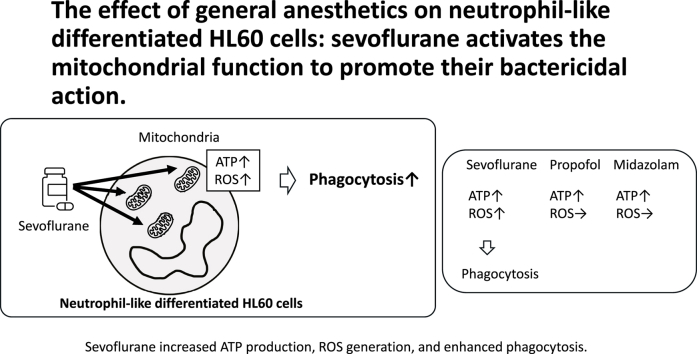

## 1 Introduction

Stress associated with surgery can reduce a patient's immunity and delay recovery after surgery. It has long been thought that general and local anesthetics used during surgery can impact immune function during the perioperative period [1]. For example, propofol and midazolam inhibit neutrophil phagocytosis, migration and production of reactive oxygen species (ROS), while lidocaine and bupivacaine do not inhibit neutrophil ROS production [[2], [3], [4]]. Sevoflurane may alter neutrophil ROS, but the results of studies are inconsistent [[5], [6], [7]]. In elective open abdominal surgery, it was shown that sevoflurane reduced the incidence of surgical site infections more than did propofol [8], but there was no difference in the incidence of surgical site infections between the two anesthetics in patients undergoing total knee replacement [9]. Therefore, the impact of anesthetics on immune cell function remains unclear. Clarifying the effects of general anesthetics and sedatives used in the perioperative period and in intensive care on immune cell function is important for promoting the recovery of the patient's general condition.

Mitochondria are intracellular organelles that are important for the functioning of immune cells and for the production of adenosine triphosphate (ATP) and ROS. ROS, which are produced as a by-product in the process of mitochondria producing energy, are highly reactive and have the risk of causing cell death through DNA damage. On the other hand, it has also been shown that ROS function as important molecules that are responsible for signal transduction, such as activation of immune cells. ROS produced from mitochondria activate immune cell function and release signal transduction molecules [10]. In addition, mitochondria themselves have a virus detection function, and it is becoming clear that mitochondrial dynamics are closely related to immune function [11]. For example, recent studies have shown that mitochondria in neutrophils have several important functions including respiratory burst, chemotaxis, NETs (neutrophil extracellular traps) formation, differentiation and death [12]. It has been shown that propofol inhibited macrophage migration, phagocytosis and ROS production by suppressing macrophage mitochondrial membrane potential and ATP synthesis [13]. Inhalation anesthetics such as sevoflurane and isoflurane act on mitochondria, suppressing mitochondrial respiration and ROS production in cardiac and brain cells [14,15]. It has been shown that general anesthetics act on the electron transport chain in mitochondria of nerve cells and inhibit the production of ROS [16]. However, it is unclear how general anesthetics affect the mitochondria of neutrophils and the function of neutrophils. In this study, we focused on the mitochondrial function of neutrophil-like differentiated HL60 cells and investigated how general anesthetics change the function of neutrophil-like differentiated HL60 cells.

## 2 Materials and methods

### 2.1 Cell culture and cell differentiation

The HL60 cell line (JCRB0085) was obtained from JCRB Cell Bank (Osaka, Japan). It was maintained in Roswell Park Memorial Institute (RPMI) 1640 medium (Thermo Fisher, Waltham, MA, USA) supplemented with 10 % fetal bovine serum (Thermo Fisher, Waltham, MA, USA) and 100 units ml^−1^ Penicillin-Streptomycin solution (Thermo Fisher, Waltham, MA, USA) at 37 °C in a 5 % CO_2_ incubator (ASTEC, Fukuoka, Japan). The cell line tested negative for mycoplasma contamination. To induce differentiation, 1 μM all-trans-Retinoic Acid (ATRA) from Fujifilm (Tokyo, Japan) was added to the medium and the cells were cultured for 4 days [17]. Cell morphology was observed using a Nikon ECLIPS 80i microscope (Nikon Corporation, Tokyo, Japan).

### 2.2 NBT (nitro blue tetrazolium) dye reduction assay

5 × 10^5^ Cells were seeded in each well of a 24-well plate and incubated with 0.1 % NBT solution in RPMI 1640 medium supplemented with 100 nM phorbol 12-myristate 13-acetate (PMA) for 30 min at 37 °C. Following incubation, the cells were centrifuged, and the resulting formazan precipitates were dissolved in dimethyl sulfoxide (DMSO). The absorbance of the solubilized formazan was measured at 570 nm using a microplate reader (Multiskan Fc, Thermo Fisher, Waltham, MA, USA). The relative absorbance values, normalized to cell number, were used as an index of differentiation efficiency.

### 2.3 Stimulation of cells using general anesthetics

The anesthetics that were tested were propofol (Maruishi Pharmaceutical Co., Ltd., Osaka, Japan), midazolam (Sandoz, Tokyo, Japan), and sevoflurane (Maruishi Pharmaceutical Co., Ltd., Osaka, Japan), based on clinical usage concentrations. HL60 cells were cultured in a medium containing 4 μg ml^−1^ propofol (Prop) [2,18] or 0.5 μg ml^−1^ midazolam (Mdz) [3,19] for 1 h or 5 h. Alternatively, the cells were exposed to 3 % sevoflurane (Sev) for 1 h or 5 h in incubators [7,20]. Unless otherwise noted, the following experiments were conducted after exposure to each anesthetic.

### 2.4 Mitochondrial activity assay

The Seahorse XFe96 extracellular flux analyzer (Agilent Technologies, Santa Clara, CA, USA) was used to measure mitochondrial oxygen consumption rate (OCR), ATP production, and maximum respiration [21]. After anesthesia, the cell medium was replaced with Seahorse analysis medium containing 10 mM glucose, 1 mM pyruvate, and 2 mM l-glutamine in pH 7.4 RPMI-1640 medium (Agilent Technologies, Santa Clara, CA, USA). Subsequently, 2 × 10^5^ cells per well were seeded into a Seahorse XFe96 well plate (Agilent Technologies, Santa Clara, CA, USA) coated with Cell Tak (Corning, NY, USA). Analysis of OCR was then performed using 1.5 μM oligomycin, 2.0 μM carbonyl cyanide 4-(trifluoromethoxy) phenylhydrazone (FCCP), 0.5 μM rotenone and 0.5 μM antimycin A following the instructions of the manufacturer (Agilent Technologies, Santa Clara, CA, USA). After measuring basal OCR, oligomycin was injected to inhibit ATP synthase, and the decrease in OCR was used to calculate ATP-linked respiration. FCCP was subsequently added to uncouple mitochondrial respiration, thereby revealing maximal respiratory capacity. Finally, rotenone and antimycin A were injected to inhibit mitochondrial complex I and III, respectively, and the remaining OCR was considered non-mitochondrial respiration. Basal respiration was defined as the baseline OCR minus non-mitochondrial respiration. ATP production was defined as the OCR reduction after oligomycin injection. Maximal respiration was defined as the OCR after FCCP injection minus non-mitochondrial respiration.

### 2.5 ROS production assay

One x 10^5^ cells were placed in each well of a 96-well plate and stained with 5 μM CellROX deep red® (Thermo Fisher, Waltham, MA, USA) following the provided instructions. To inhibit mitochondrial respiration, we used a solution containing 0.5 μM rotenone and 0.5 μM antimycin A (Rot/AA) from Sigma Aldrich (USA) before anesthesia [22,23]. After staining, the cells were washed three times with Hank's Balanced Salt Solution (HBSS) and analyzed using the All-in-One Fluorescence Microscope BZ-X700 (BZ-X700) from KEYENCE (Osaka, Japan). To analyze the data, ImageJ software (NIH, USA) was used to compare fluorescence intensities [24]. Fifty CellROX deep red-positive cells were selected to assess the average fluorescence brightness. All measurements were expressed relative to the untreated control for each experiment.

### 2.6 Apoptosis assay

We used the Annexin V-FITC/PI assay kit (MBL, Tokyo, Japan) according to the manufacturer's protocol. The cells were stained with annexin V and propidium iodide (PI) for 15 min at room temperature in the dark. The cells were subsequently analyzed using the BZ-X700 microscope along with BZ-X800 analysis software. To evaluate cell death, we calculated the percentage of cells that were positive for annexin V, PI, or both relative to the total number of cells [25].

### 2.7 Cell viability and cytotoxicity assay

Cell viability was assessed by morphological assessment under bright-field microscopy; cells with intact membranes and refractile appearance were scored as viable. Cell viability was calculated by dividing the number of viable cells by the total number of cells. Cell viability and Cytotoxicity were measured using the MTT assay (Premix WST-1 Cell Proliferation Assay System, Takara Bio Inc, Shiga, Japan) that utilizes a reagent to measure cell proliferation and viability through chromogenic analysis. Specifically, the tetrazolium salt (WST-1) is converted into formazan dye by the mitochondrial dehydrogenase present in living cells. We seeded 4 × 10^4^ cells per well in a 96-well plate and added the MTT reagent to each well. After 2-h incubation at 37 °C in a 5 % CO_2_ incubator, we measured the sample with a microplate reader (Multiskan Fc, Thermo Fisher, Waltham, MA, USA). Cell viability was calculated by comparing the absorbance of the control cells to that of the treated cells.

### 2.8 pHrodo bioparticles phagocytosis assay

We used pHrodo Bioparticles to analyze the impact of anesthetics on the bactericidal activity associated with phagocytosis of differentiated HL60 cells. First, we seeded 0.8 × 10^6^ cells per well in a 6-well plate and cultured them with 0.5 μg ml^−1^ pHrodo red Zymosan A Bioparticles (Thermo Fisher, Waltham, MA, USA) during anesthesia. Then we collected the cells and stained them with Hoechst 33342 (DOJINDO, Kumamoto, Japan) for nuclear staining. We captured fluorescence images using the BZ-X700 microscope and performed analysis with BZ-X800 software. The beads are phagocytosed and exposed to intracellular low pH lysosomes, which fluoresce only under acidic conditions, causing the pHrodo to emit red fluorescence. We calculated the percentage of red particles to DAPI-positive cells to determine the amount of phagocyted particles [26].

### 2.9 Mitochondrial morphology

Cells were placed in 96-well plates and stained with Mito Bright LT Green probes (DOJINDO, Kumamoto, Japan, excitation: 488 nm, emission: 500–560 nm) and Hoechst 33342 solution (excitation: 352 nm, emission: 461 nm) according to the manufacturer's instructions. Fluorescent images were captured using the BZ-X700 microscope. Fluorescent microscope filters were as follows: GFP filter (excitation: 470/40, emission: 525/50) for Mito Bright LT Green and DAPI filter (excitation: 360/40, emission: 460/50) for Hoechst 33342. The intensity of Mito Bright LT Green fluorescence was measured using imageJ, and the level of mitochondrial expression was evaluated. Fifty Mito Bright LT Green-positive cells were selected to assess the average fluorescence Intensity. We used the LSM700 confocal laser microscope (Carl Zeiss, Jena, Germany) to observe the mitochondria in more detail. Excitation wavelengths of 488 nm for Mito Bright LT Green and 405 nm for Hoechst 33342 as well as appropriate filters were used. After staining, cell fluid was sealed on a glass slide for observation. We quantified the morphology of mitochondria by measuring the aspect ratio (length-to-width ratio) using ImageJ [27].

### 2.10 Statistical analysis

**Figure 1:**
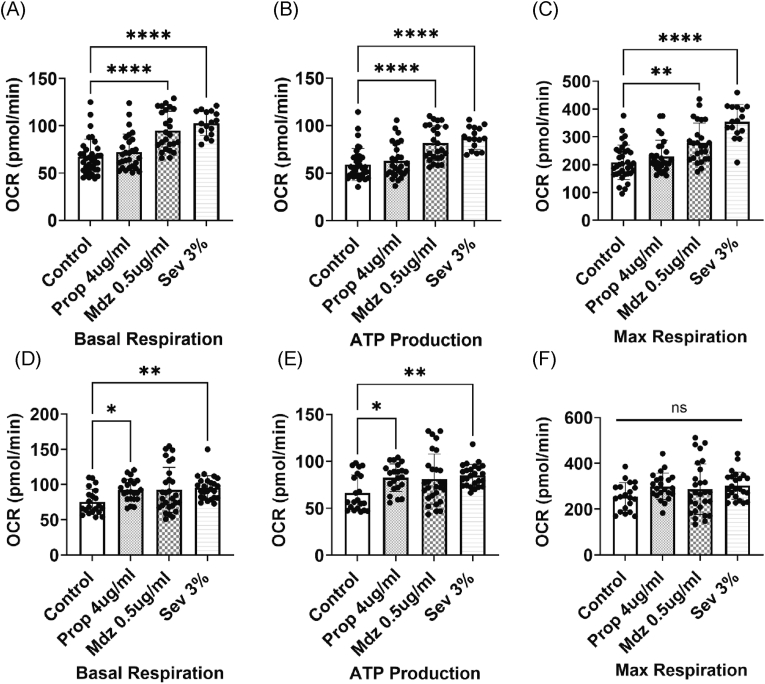
General anesthetics activated mitochondrial respiration in differentiated HL60 cells. The measured parameters include basal respiration (A, D), ATP production (B, E), and maximum respiration (C, F) after 1 h and 5 h of anesthesia. Each bar in the graphs represents the mean ± standard deviation. Independent experiments were performed three times. Replicate wells per experiment was n = 4–10. To reduce variability, the data from all experiments were analyzed collectively. Analysis was performed by using the Kruskal-Wallis test followed by Dunn's multiple comparison test. Statistical significance is indicated as follows: ∗p < 0.05, ∗∗p < 0.01, ∗∗∗p < 0.001, ∗∗∗∗p < 0.0001.

**Fig. 2:**
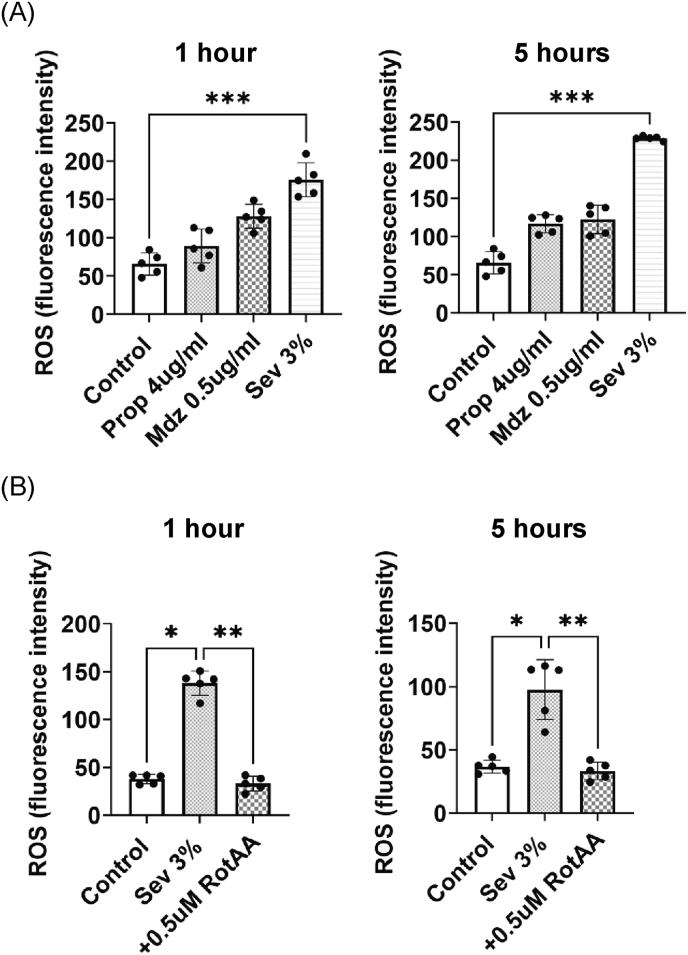
General anesthetics affect the production of reactive oxygen species (ROS) in differentiated HL60 cells. The production of ROS was assessed after 1 h and 5 h of anesthesia (A). The increase in the production of ROS caused by 3 % sevoflurane anesthesia was reduced by mitochondrial respiratory inhibitors, rotenone and antimycin (Rot/AA) (B). Each bar in the graphs represents the mean ± standard deviation. Independent experiments were performed three times. Replicate wells per experiment was n = 5. Analysis was performed by using the Kruskal-Wallis test followed by Dunn's multiple comparison test. Statistical significance is indicated as follows: ∗p < 0.05, ∗∗p < 0.01, ∗∗∗p < 0.001.

**Fig. 3:**
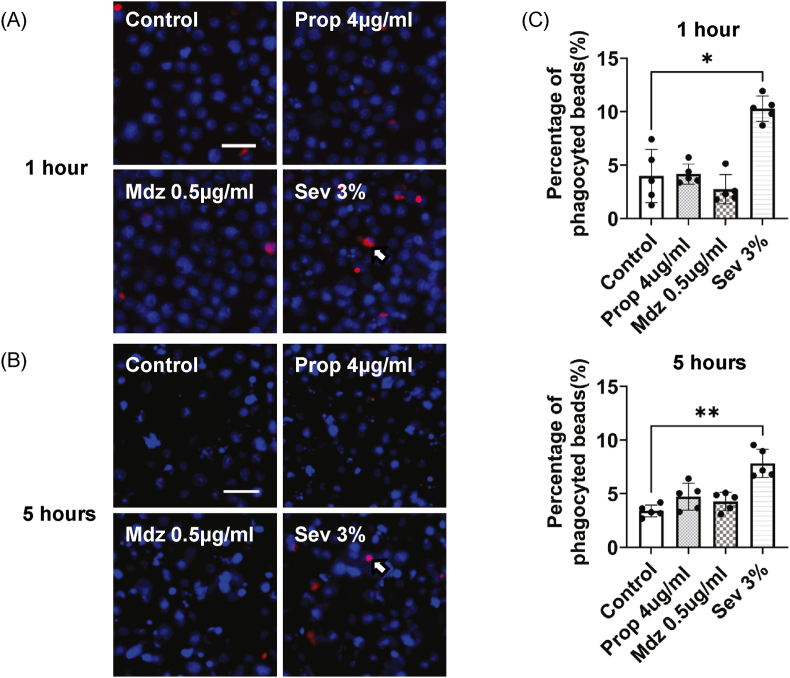
The bactericidal effect of ROS was investigated by examining phagocytosis rates in the presence of general anesthetics. Sevoflurane significantly increased bactericidal activity compared to that in the control groups at both 1 h and 5 h of anesthesia (A–C). Red coloring indicates ROS-producing cells related to phagocytosis (white arrow). Blue indicates the nucleus. The white bar in the image represents a scale of 20 μm (A, B). Each bar represents the mean ± standard deviation. Independent experiments were performed three times. Replicate wells per experiment was n = 5. Analysis was performed by using the Kruskal-Wallis test followed by Dunn's multiple comparison test. Statistical significance is indicated as ∗p < 0.05, ∗∗p < 0.01.

**Fig. 4:**
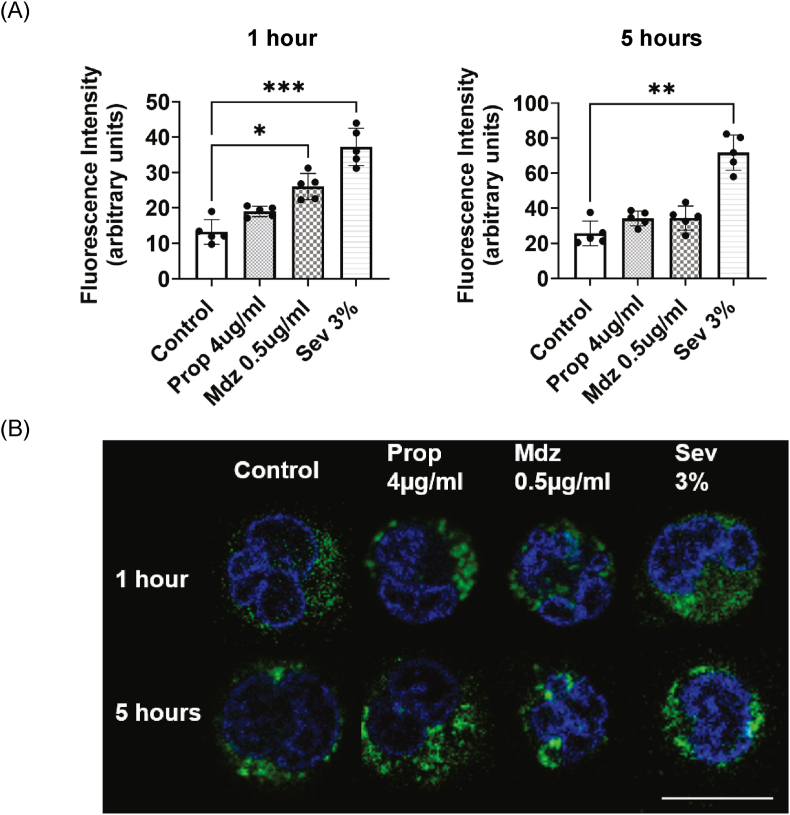
Mitochondrial morphology after 1 h and 5 h of anesthesia. The impact of anesthetics on mitochondrial expression was assessed using the average GFP fluorescence intensity (A). Each bar represents the mean ± standard deviation. Independent experiments were performed twice. Replicate wells per experiment was n = 5. Analysis was performed by using the Kruskal-Wallis test followed by Dunn's multiple comparison test. Statistical significance is indicated as ∗p < 0.05, ∗∗p < 0.01, ∗∗∗p < 0.001. Filamentous mitochondria were observed in the anesthesia groups, but fragmented mitochondria were present in the control group. Green indicates mitochondria and blue indicates the nucleus. The white bar represents 10 μm (B).

Each independent experiment was repeated at least twice. Independent experiments were performed three times for Figure 1, Fig. 2, Fig. 3 and Supplementary Figs. 2–4, and twice for Fig. 4 and Supplementary Figs. 1, 5, 6. Replicate wells per experiment were n = 4–10 (Fig. 1, Supplementary Fig. S2), n = 5 (Fig. 2, Fig. 3, Fig. 4, Supplementary Fig. S3), n = 6 (Supplementary Fig. S1), n = 4–5 (Supplementary Figs. S4 and S5), and n = 10 (Supplementary Fig. S6). To reduce variability, the data from all experiments were analyzed collectively (Fig. 1). Data are presented as means ± standard deviation. We analyzed the differences between the results using the Kruskal-Wallis test followed by Dunn's multiple comparison test and the Mann-Whitney *U* test in Prism10™ (GraphPad Software, San Diego, CA, USA). A p-value of less than 0.05 was considered statistically significant.

## 3 Results

### 3.1 Differentiated HL60 cell morphology

HL60 cells were induced to differentiate into neutrophil-like HL60 cells using ATRA. The differentiated HL60 cells exhibited neutrophil-like morphology and had a divided nucleus (Supplementary Fig. S1 A, B)

### 3.2 NBT assay confirming HL60 cell differentiation into neutrophil-like cells

The differentiation efficiency of HL60 cells was evaluated using the NBT assay. Differentiated HL60 cells exhibited increased NBT reductive activity compared with the controls. (Supplementary Fig. S1 C).

### 3.3 General anesthetics alter mitochondrial metabolism in differentiated HL60 cells

A representative Seahorse XFe96 extracellular flux analyzer trace is shown to illustrate the OCR profile during the assay (Supplementary Fig. S2). Midazolam and sevoflurane significantly activated mitochondrial respiration, resulting in increased ATP production after 1 h of anesthesia (Fig. 1A–C). Propofol and sevoflurane also activated mitochondrial respiration and significantly boosted ATP production after 5 h of anesthesia (Fig. 1D and E). However, none of the anesthetics changed the maximum respiratory rate after 5 h of anesthesia (Fig. 1 F).

### 3.4 General anesthetics affect production of ROS in differentiated HL60 cells

Propofol and midazolam did not increase the production of ROS after either 1 h or 5 h (Fig. 2 A, Supplementary Fig. S3 A, B). Sevoflurane significantly increased the production of ROS at both 1 h and 5 h. The increase in the production of ROS caused by 3 % sevoflurane anesthesia was significantly reduced by mitochondrial respiratory inhibitors (Fig. 2 B).

### 3.5 None of the anesthetic had any cytotoxic effects on differentiated HL60 cells

Propofol, midazolam, and sevoflurane did not increase apoptosis of differentiated HL60 cells compared with controls by 1-h and 5-h exposure (Supplementary Fig. S4 A-C). In addition, none of the anesthetics caused a significant decrease in cell activity or an increase in the number of dead cells during the 1-h and 5-h exposure periods (Supplementary Fig. S5 A-C).

### 3.6 Sevoflurane significantly increased bactericidal activity by phagocytosis

Exposure of differentiated HL60 cells to sevoflurane significantly enhanced the phagocytosis of fluorescent beads compared to that in the control group. In contrast, exposure to propofol or midazolam did not alter the phagocytic activity of the cells (Fig. 3A–C).

### 3.7 Mitochondrial morphology of differentiated HL60 cells after exposure to anesthetics

Exposure to sevoflurane for 1 h and 5 h and exposure to midazolam for 1 h enhanced the fluorescence of differentiated HL60 cells, as indicated by Mito Bright LT Green staining (Fig. 4 A, Supplementary Fig. S6 A, B). The evaluation of mitochondrial morphology revealed that fragmented and filamentous mitochondria were present in the control group, whereas filamentous mitochondria were observed in the groups treated with propofol, midazolam, and sevoflurane (Fig. 4 B). Quantitative analysis showed that the mitochondrial aspect ratio ranged from 1.5 to 2 on average, and there were no significant differences between the control group and any of the anesthetic-treated groups (Supplementary Fig. S6 C).

## 4 Discussion

This study showed that the general anesthetics propofol, midazolam, and sevoflurane activate mitochondrial function in differentiated HL60 cells. Propofol and midazolam partially activated mitochondrial function but did not increase the production of ROS or phagocytosis. Sevoflurane was shown to promote the production of ROS in neutrophil-like differentiated HL60 cells and to promote phagocytosis, which is one of the bactericidal effects (see Table 1).

**Table 1:** Summary of changes in mitochondrial respiration, ATP production, ROS levels, and phagocytosis in differentiated HL60 cells assessed after 1 h and 5 h of anesthesia with each anesthetic.

		Prop 4 μg ml^−1^	Mdz 0.5 μg ml^−1^	Sev 3 %
Mitochondrial respiration	1 h	(−)	**⇑**	**⇑**
	5 h	**⇑**	(−)	**⇑**
ATP production	1 h	(−)	**⇑**	**⇑**
	5 h	**⇑**	(−)	**⇑**
ROS levels	1 h	(−)	(−)	**⇑**
	5 h	(−)	(−)	**⇑**
Phagocytosis	1 h	(−)	(−)	**⇑**
	5 h	(−)	(−)	**⇑**

(−), not changed; **⇑,** increased; Prop, Propofol; Mdz, Midazolam; Sev, Sevoflurane; ATP, adenosine triphosphate; ROS, reactive oxygen species.

Research on the immune system using human neutrophils has been conducted for many years. However, there have been concerns that neutrophils have a short lifespan and are likely to be subject to unexpected external stimuli [28]. On the other hand, the cells used in this study were differentiated HL60 cells. HL60 cells are human promyelocytic leukemia cells that can be differentiated into neutrophil-like cells by ATRA [29]. The NBT assay confirmed successful differentiation of HL60 cells into neutrophil-like cells. Differentiated HL60 cells have a longer lifespan than neutrophils of biological origin and are less susceptible to external factors, making them suitable for *in vitro* experiments involving prolonged exposure to anesthetics [30]. In this study, we investigated the effects of general anesthetics on the function of differentiated HL60 cells.

There have been many studies in which the effects of general anesthetics on immune cells such as neutrophils were investigated [[1], [2], [3], [4], [5]], but there have been few studies focusing on mitochondrial function. Mitochondria are important organelles in neutrophils that are involved in respiratory burst, chemotaxis, NETs (neutrophil extracellular traps) formation, differentiation and death [31]. ROS produced following mitochondrial activation are generally an indicator of cytotoxicity, but in neutrophils, they are also used for the bactericidal effect against foreign pathogens [32]. In this study, exposure to propofol, midazolam and sevoflurane significantly increased mitochondrial respiration and ATP production in differentiated HL60 cells, indicating mitochondrial activation. However, only sevoflurane significantly increased the production of ROS. Although propofol and midazolam did not significantly increase the production of ROS compared to the control group in these experiments, it is possible that exposure to anesthetics may increase the production of ROS in the same way as that in the case of exposure to sevoflurane depending on the conditions of exposure to the anesthetic. In this study, propofol and midazolam were administered as a single dose to the cell culture medium at the target concentration, and it is therefore possible that the anesthetic agents were partially inactivated or metabolized during exposure and the concentration of the anesthetic agents decreased. Alternatively, it is possible that the energy produced by the activation of mitochondria during exposure to sevoflurane is used for the bactericidal effects mediated by reactive oxygen species instead of exposure to propofol and midazolam [22,33,34].

On the other hand, no cytotoxicity or apoptosis was observed with sevoflurane, suggesting that the ROS produced by sevoflurane were not toxic to the cells themselves. In experiments using pHrodo bioparticles, sevoflurane significantly increased the bactericidal effect associated with phagocytosis. These results suggest that an increase in the production of ROS by sevoflurane may be related to its bactericidal action against pathogens. It was shown that sevoflurane was less likely than propofol to increase plasma endotoxin levels in sepsis model mice, suggesting that it improves mouse survival [35], which may be an effect of sevoflurane promoting neutrophil ROS production in *in vitro* models [6]. Further research is needed to determine whether the use of sevoflurane to promote the bactericidal action of neutrophils during general anesthesia can reduce the risk of perioperative infection or improve the prognosis of patients with sepsis.

Production of ROS can occur via two pathways: one mediated by nicotinamide adenine dinucleotide phosphate (NADPH) in the cytoplasm and the other mediated via the mitochondrial electron transport chain [36]. Antimycin A inhibits the electron transport complex III (cytochrome *c* reductase), significantly reducing neutrophil superoxide O_2_^−^ production [36]. This suggests that the production of ROS in neutrophils is mainly regulated by mitochondria. In this study, the increase in the production of ROS caused by sevoflurane was suppressed by a mitochondrial respiratory inhibitor, suggesting that sevoflurane may have increased the production of ROS by acting on the mitochondrial respiratory chain complexes I and III.

Healthy mitochondria repeatedly undergo fusion and fission over time. We observed that the fluorescence intensity of mitochondria increased after 1 h of exposure to midazolam, 1 h of exposure to sevoflurane and 5 h of exposure to sevoflurane. These results support the results of our research showing that exposure to midazolam for 1 h and exposure to sevoflurane for 1 h and 5 h activate mitochondrial function in HL60 cells. Morphologically, fragmented or filamentous mitochondria indicate a healthy state, and spherical mitochondria indicate a damaged state due to ROS [37]. Since spherical mitochondria were not observed in any of the conditions, the morphology of the mitochondria suggests that they may be healthy rather than in a stressed state.

## 5 Limitations

This study showed that sevoflurane activates mitochondrial function in neutrophil-like differentiated HL60 cells and promotes their bactericidal action, but the underlying molecular mechanisms have not yet been elucidated. In the future, if it becomes clear where the action site of sevoflurane is and the mechanism of activation of neutrophil function becomes clear, it is hoped that an anesthetic that contributes to infection control can be developed. In addition, we conducted an anesthetic exposure experiment using the neutrophil-like cultured cell line HL60, but the results obtained in this study may be influenced by the type of cell, the differentiation culture conditions [17], or the exposure time and concentration of the anesthetics. Although we were able to observe the direct effects of anesthetics on cell function *in vitro*, *in vivo* anesthetic exposure may cause neutrophil-like differentiated HL60 cells to exhibit different kinetics due to the influence of other factors. Furthermore, differentiated HL60 cells may have lower phagocytic activity and higher mitochondrial content compared to mature neutrophils, leading to increased OCR. Therefore, the findings of this study may not be directly applicable to mature neutrophils, and further studies using isolated neutrophils will be necessary.

Another limitation of this study is the relatively small number of independent experiments. Although each assay was repeated at least twice with multiple technical replicates, the limited number of biological replicates may reduce the robustness of the statistical analyses. Future studies with a larger number of independent experiments will be required to confirm and strengthen these findings.

## 6 Conclusion

The results of this study indicate that exposure to sevoflurane activates mitochondrial function in differentiated HL60 cells and leads to the production of ROS and promotes their bactericidal action. The results of this study may provide promising insights into the development of effective approaches for preventing complications associated with a decline in immune function during the perioperative period.

## CRediT authorship contribution statement

**Kosuke Asei:** Writing – original draft, Visualization, Software, Methodology, Investigation, Formal analysis, Data curation, Conceptualization. **Yuki Nomura:** Writing – review & editing, Writing – original draft, Visualization, Validation, Supervision, Software, Resources, Methodology, Investigation, Formal analysis, Conceptualization. **Daichi Fujimoto:** Supervision, Software, Resources, Investigation, Formal analysis, Data curation. **Mayu Ooi:** Supervision, Resources, Funding acquisition, Conceptualization. **Norihiko Obata:** Supervision, Resources, Formal analysis. **Satoshi Mizobuchi:** Writing – review & editing, Supervision, Formal analysis, Conceptualization.

## Declaration of competing interest

The authors declare that they have no known competing financial interests or personal relationships that could have appeared to influence the work reported in this paper.

## Data Availability

Data will be made available on request.

## References

[1] Ackkerman R.S., Luddy K.A., Icard B.E., Fernández J.P., Gatenby R.A., Muncey A.R. (2021). The effects of anaesthetics and perioperative medications on immune function. Anesth. Analg..

[2] Mikawa K., Akamatsu H., Nishina K., Shiga M., Maekawa N., Obara H., Niwa Y. (1998). Propofol inhibits human neutrophil functions. Anesth. Analg..

[3] Nishina K., Akamatsu H., Mikawa K., Shiga M., Maekawa N., Obara H., Niwa Y. (1998). The inhibitory effects of thiopental, midazolam, and ketamine on human neutrophil functions. Anesth. Analg..

[4] Kolle G., Metterlein T., Gruber M., Seyfried T., Petermichl W., Pfaehler S.M., Bredthauer Andre (2021). Potential impact of local anesthetics inducing granulocyte arrest and altering immune functions on perioperative outcome. J. Inflamm. Res..

[5] Fahlenkamp A.V., Coburn M., Rossaint R., Stoppe C., Haase H. (2014). Comparison of the effects of xenon and sevoflurane anaesthesia on leucocyte function in surgical patients. Br. J. Anaesth..

[6] Minguet G., Franck T., Joris J., Serteyn D. (2017). Sevoflurane modulates the release of reactive oxygen species, myeloperoxidase, and elastase in human whole blood. Int. J. Immunopathol. Pharmacol..

[7] Strosing K.M., Faller S., Gyllenram V., Engelstaedter H., Buerkle H., Spassov S., Hoetzel A. (2016). Inhaled anesthetics exert different protective properties in a mouse model of ventilator-induced lung injury. Anesth. Analg..

[8] Shimizu K., Hirose M., Mikami S., Takamura K., Goi T., Yamaguchi A., Shigemi K. (2010). Effect of anaesthesia maintained with sevoflurane and propofol on surgical site infection after elective open gastrointestinal surgery. J. Hosp. Infect..

[9] Kishimoto M., Yamana H., Inoue S., Noda T., Akahane M., Inagaki Y., Imamura T. (2018). Suspected periprosthetic joint infection after total knee arthroplasty under Propofol versus Sevoflurane Anesthesia. Can. J. Anaesth..

[10] Angajala A., Lim S., Phillip J.B., Kim J.H., Yates C., You Z., Tan M. (2018). Diverse roles of mitochondria in immune responses. Front. Immunol..

[11] Hanada Y., Ishihara N., Wang L., Otera H., Ishihara T., Koshiba T., Nomura M. (2020). MAVS is energized by Mff which senses mitochondrial metabolism via AMPK for acute antiviral immunity. Nat. Commun..

[12] Cao Z., Zhao M., Sun H., Hu L., Chen Y., Fan Z. (2022). Roles of mitochondria in neutrophils. Front. Immunol..

[13] Chen R.M., Wu C.H., Chang H.C., Wu G.J., Lin Y.L., Sheu J.R., Chen T.L. (2003). Propofol suppresses macrophage functions and modulates mitochondrial membrane potential and cellular adenosine triphosphate synthesis. Anesthesiology.

[14] Orriach J.L.G., Luque M.D.C., Ponferrada A.R. (2023). Beneficial effects of halogenated anesthetics in cardiomyocytes. Antioxidants.

[15] Archer D.P., Walker A.M., McCann S.K., Moser J.J., Appireddy R.M. (2017). Anesthetic neuroprotection in experimental stroke in rodents. Anesthesiology.

[16] Ye R., Yang Q., Kong X., Li N., Zhang Y., Han J., Zhao G. (2012). Sevoflurane preconditioning improves mitochondrial function and long-term neurologic sequelae after transient cerebral ischemia. Crit. Care Med..

[17] Tasseff R., Jensen H.A., Congleton J., Dai D., Rogers K.V., Sagar A., Varner J.D. (2017). An effective model of the retinoic acid induced HL-60 differentiation program. Sci. Rep..

[18] Twaroski D.M., Yan Y., Zaja I., Clark E., Bosnjak Z.J., Bai X. (2015). Altered mitochondrial dynamics contributes to propofol-induced cell death in human stem cell-derived neurons. Anesthesiology.

[19] Weiss M., Mirow N., Birkhahn A., Schneider M., Wernet P. (1993). Benzodiazepines and their solvents influence neutrophil granulocyte function. Br. J. Anaesth..

[20] Han X.C., Zhang Y.J., Dong X., Xing Q.Z., Li K.H., Zhang L. (2020). Sevoflurane modulates the cancer stem cell-like properties and mitochondrial membrane potential of glioma via Ca2+-dependent CaMKII/JNK cascade. Life Sci..

[21] Yoo I., Ahn I., Lee J., Lee N. (2024). Extracellular flux assay (Seahorse Assay): diverse applications in metabolic research across biological disciplines. Mol. Cell.

[22] Chen Q., Vazquez E.J., Moghaddas S., Hopple C.L., Lesnefsky E.J. (2003). Production of reactive oxygen species by mitochondria. J. Biol. Chem..

[23] Griffiths L.A., Flatters S.J.L. (2015). Pharmacological modulation of the mitochondrial electron transport chain in paclitaxel-induced painful peripheral neuropathy. J. Pain.

[24] Rizzi E., Guimaraes D.A., Ceron C.S., Prado C.M., Pinheiro L.C., Oliveira A.M., Santos J.E.T. (2014). Β1-adrenergic blockers exert antioxidant effects, reduce matrix metalloproteinase activity, and improve renovascular hypertension-induced cardiac hypertrophy. Free Radic. Biol. Med..

[25] Asano M., Tanaka S., Sakaguchi M., Matsumura H., Yamaguchi T., Fujita Y., Tabuse K. (2017). Normothermic microwave irradiation induces death of HL-60 cells through heat-independent apoptosis. Sci. Rep..

[26] Lindner B., Burkard T., Schuler M. (2020). Phagocytosis assays with different pH-sensitive fluorescent particles and various readouts. Biotechniques.

[27] Koopman W.J.H., Visch H.J., Smeitink J.A.M., Willems P.H.G.M. (2006). Simultaneous quantitative measurement and automated analysis of mitochondrial morphology, mass, potential, and motility in living human skin fibroblasts. Cytometry. A..

[28] Hidalgo A., Chilvers E.R., Summers C., Koenderman L. (2019). The neutrophil life cycle. Trends Immunol..

[29] Nordenfelt P., Bauer S., Lönnbro P., Tapper H. (2009). Phagocytosis of streptococcus pyogenes by all-trans retinoic acid-differentiated HL-60 cells: roles of azurophilic granules and NADPH oxidase. PLoS One.

[30] Dong X., Peng S., Ling Y., Huang B., Tu W., Sun X., Wu J. (2023). ATRA treatment slowed P-selectin-mediated rolling of flowing HL60 cells in a mechano-chemical-dependent manner. Front. Immunol..

[31] Cao Z., Zhao M., Sun H., Hu L., Chen Y., Fan Z. (2022). Roles of mitochondria in neutrophils. Front. Immunol..

[32] Dahlgren C., Karlsson A., Bylund J. (2019). Intracellular neutrophil oxidants. J. Immunol..

[33] Serrano M.M., Pardo M.G., Monsalve A.M., Sánchez M.D.C. (2017). Antibacterial effect of sevoflurane and isoflurane. Rev. Esp. Quimioter..

[34] Li W.X., Tong X., Yang P.P., Zhang Y., Liang J.H., Li G.H., Dai S.X. (2022). Screening of antibacterial compounds with novel structure from the FDA approved drugs using machine learning methods. Aging (Albany NY).

[35] Schläpfer M., Piegeler T., Dull R.O., Schwartz D.E., Mao M., Bonini M.G., Minshall R.D. (2015). Propofol increases morbidity and mortality in a rat model of sepsis. Crit. Care.

[36] Snary K.J.D., Surewaard B.G., Mewburn J.D., Bentley R.E., Martin A.Y., Jones O., Archer S.L. (2022). Mitochondria in human neutrophils mediate killing of Staphylococcus aureus. Redox Biol..

[37] Willems P.H.G.M., Rossignol R., Dieteren C.E.J., Murphy M.P., Koopman W.J.H. (2015). Redox homeostasis and mitochondrial dynamics. Cell. Metab..

