# Supplementary material for: The effect of general anesthetics on neutrophil-like differentiated HL60 cells: sevoflurane activates the mitochondrial function to promote their bactericidal action
Source: Biochem Biophys Rep. 2025 Sep 19;44:102272. doi: 10.1016/j.bbrep.2025.102272 (PMC12477849; doi:10.1016/j.bbrep.2025.102272)
Supplement: Multimedia component 1 [file mmc1.docx]

Supplementary figure

Supplementary Figure S1. HL60 cell (A). Differentiated HL60 cell (B). The nucleus appears lobulated, and the cell exhibits a neutrophil-like morphology. The black bar in the image represents 10 µm. Differentiation efficiency was quantified using the NBT dye reduction assay (C) Differentiated HL60 cells exhibited increased NBT reductive activity compared with the controls.

Each bar in the graphs represents the mean ± standard deviation. Independent experiments were performed twice. Replicate wells per experiment was n=6. Analysis was performed by using the Mann-Whitney U test. Statistical significance is indicated as follows: **p < 0.01.

Supplementary Figure S2. The definitions of basal respiration, ATP production, and maximal respiration are illustrated in the diagram (A). Representative Seahorse traces corresponding to the presented OCR data are shown, with panel B representing the 1 hour time point and panel C the 5 hour time point (B, C). Independent experiments were performed three times. Replicate wells per experiment was n=4-10.

Supplementary Figure S3. Effects of anesthetic drugs on reactive oxygen species (ROS) in differentiated HL60 cells at 1 hour and 5 hours (A, B). Red coloring indicates ROS-producing cells (white arrow). The white bar in the images represents a scale of 50 µm. Independent experiments were performed three times. Replicate wells per experiment was n=5.

Supplementary Figure S4. Cell apoptosis after 1 hour and 5 hours of anesthesia. The percentage of cell death was calculated as the number of annexin V-, PI-, or both annexin V and PI-positive cells divided by the total number of cells at 1 hour (A, C) and 5 hours (B, C). Although the overall apoptosis and dead cell count increased after 5 hours of anesthesia, the total number of cells also increased, resulting in a consistent ratio. Green indicates annexin V-positive cells (white arrow), and red indicates PI-positive cells (black arrowhead). The white bar in the image represents 50 µm. Each bar represents the mean ± standard deviation. Independent experiments were performed three times. Replicate wells per experiment was n=4-5. Analysis was performed by using the Kruskal-Wallis test followed by Dunn's multiple comparison test. “ns” represents not significant.

Supplementary Figure S5. Cell viability was assessed by morphological assessment under bright-field microscopy; cells with intact membranes and refractile appearance were scored as viable. Cell viability was calculated by dividing the number of viable cells by the total number of cells (A, B). To validate this approach, a subset of samples was also analyzed by MTT assay according to the manufacturer’s protocol (C). The white bar in the image represents 50 µm. Each bar represents the mean ± standard deviation. Independent experiments were performed twice. Replicate wells per experiment was n=4-5. Analysis was performed by using the Kruskal-Wallis test followed by Dunn's multiple comparison test. “ns” represents not significant.

Supplementary Figure S6. Mitochondrial morphology after 1 hour and 5 hours of anesthesia (A, B). Green indicates mitochondria (white arrow) and blue indicates the nucleus. The white bar represents 20 µm. Mitochondrial aspect ratio in differentiated HL-60 cells after 1 and 5 hours of anesthetic exposure (C). The aspect ratio (length-to-width ratio) of mitochondria was measured using ImageJ. Each bar represents the mean ± standard deviation. Independent experiments were performed twice. Replicate wells per experiment was n=10. Analysis was performed by using the Kruskal-Wallis test followed by Dunn's multiple comparison test. “ns” represents not significant.
