# Supplementary figures and images for: The effect of general anesthetics on neutrophil-like differentiated HL60 cells: sevoflurane activates the mitochondrial function to promote their bactericidal action
Source: Biochem Biophys Rep. 2025 Sep 19;44:102272. doi: 10.1016/j.bbrep.2025.102272 (PMC12477849; doi:10.1016/j.bbrep.2025.102272)

# Supplementary Figure S1

(A)

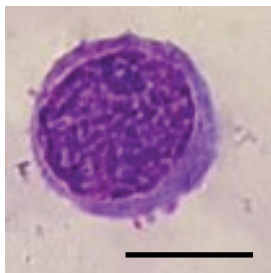

(B)

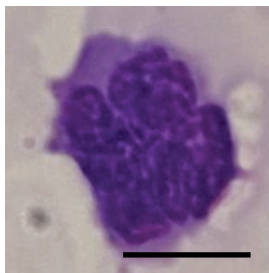

(C)

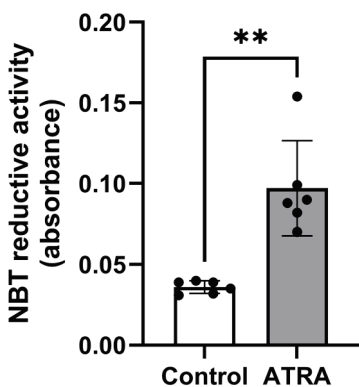

Supplement: Multimedia component 2 [file mmc2.pdf]

# Supplementary Figure S2

(A)

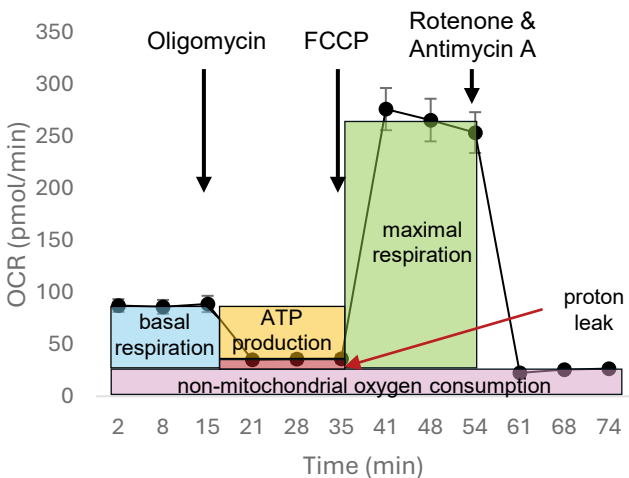

(B)

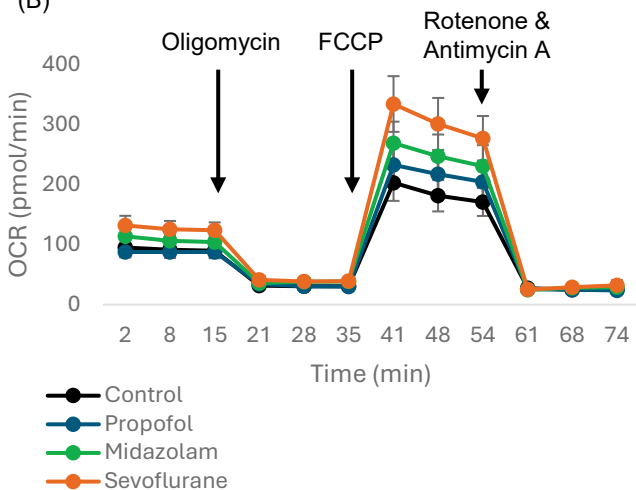

(C)

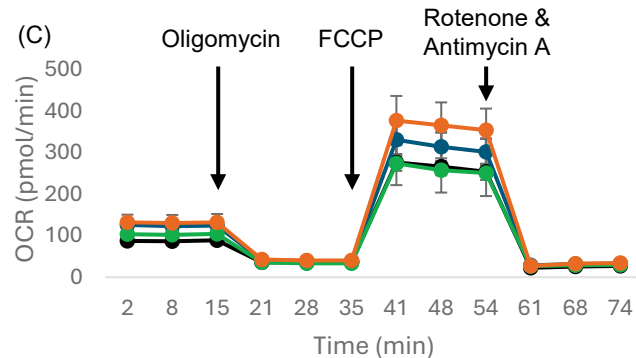

Supplement: Multimedia component 3 [file mmc3.pdf]

# Supplementary Figure S3

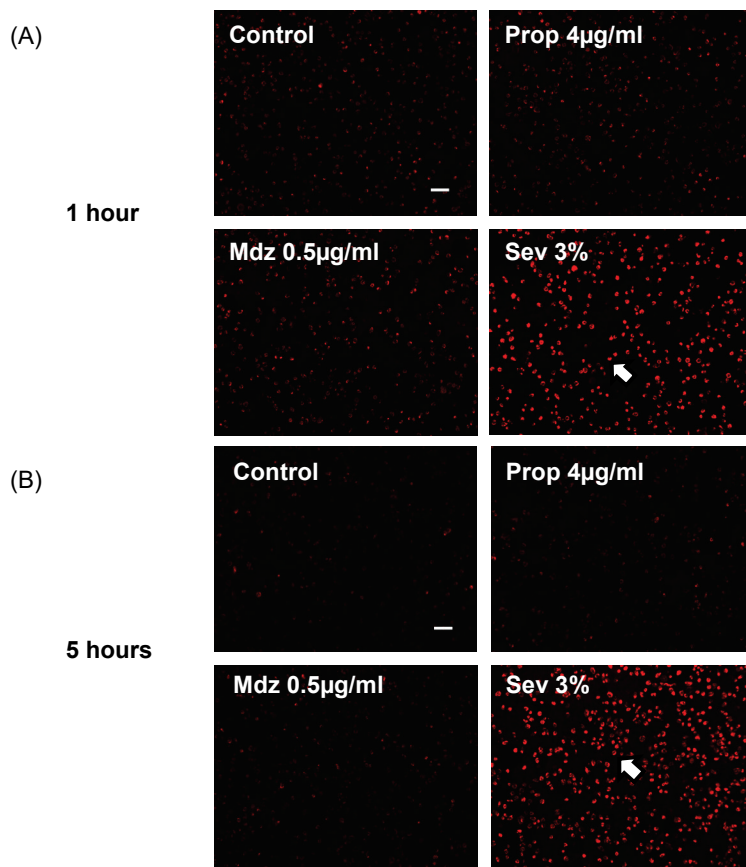

Supplement: Multimedia component 4 [file mmc4.pdf]

# Supplementary Figure S4

(A)

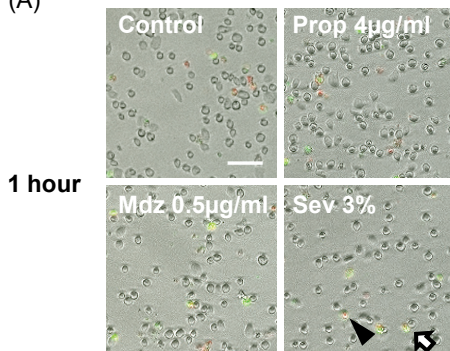

(B)

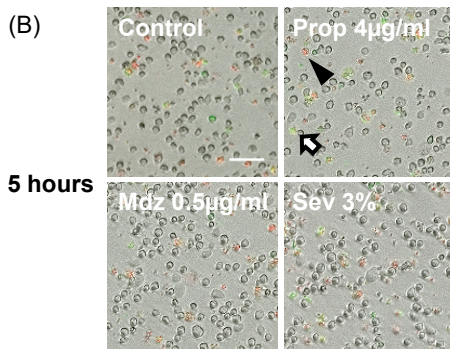

(C)

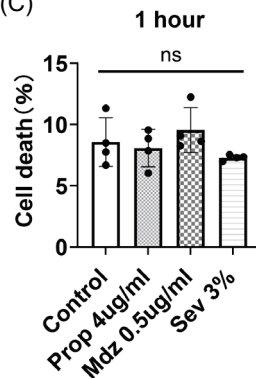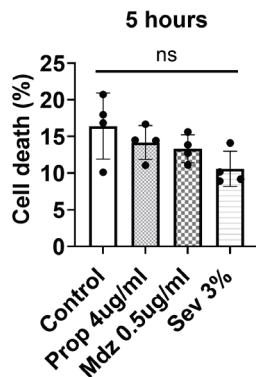

Supplement: Multimedia component 5 [file mmc5.pdf]

# Supplementary Figure S5

(A)

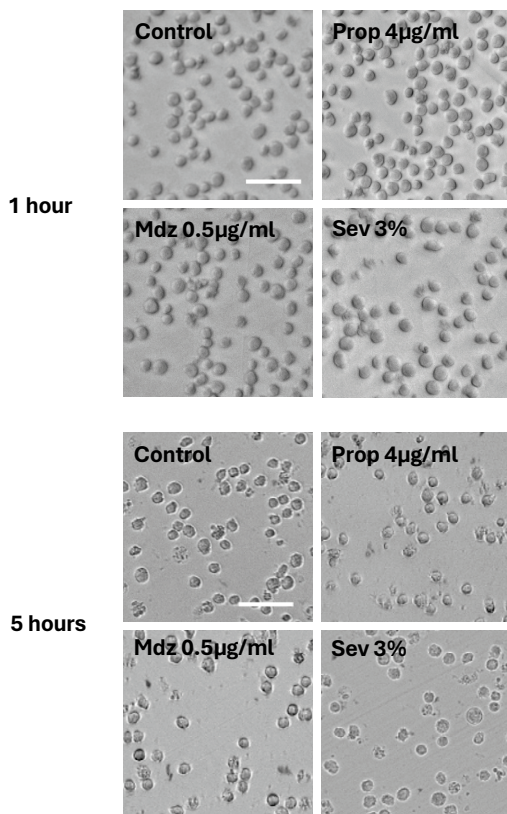

(B)

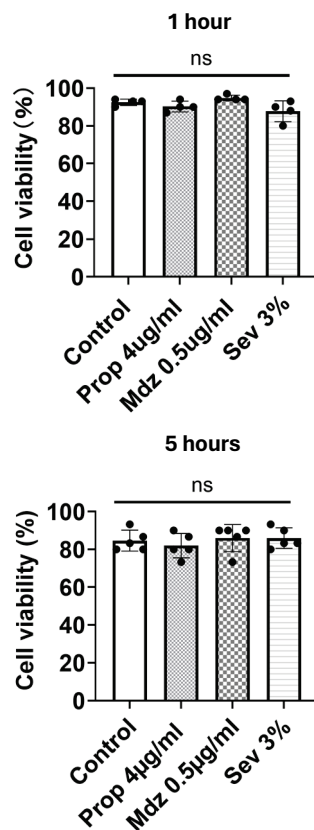

(C)

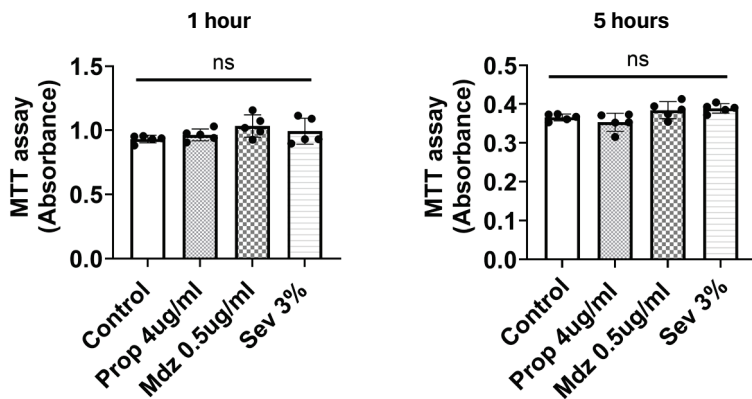

Supplement: Multimedia component 6 [file mmc6.pdf]

# Supplementary Figure S6

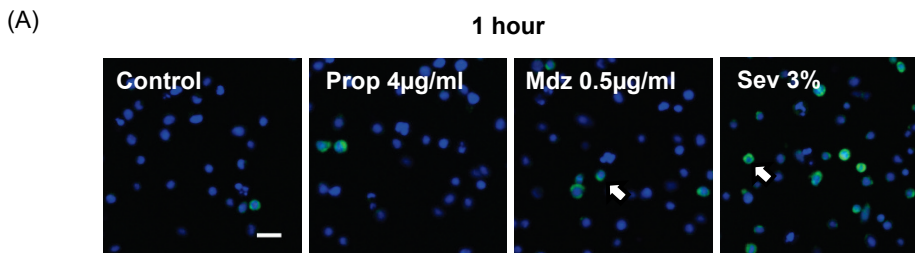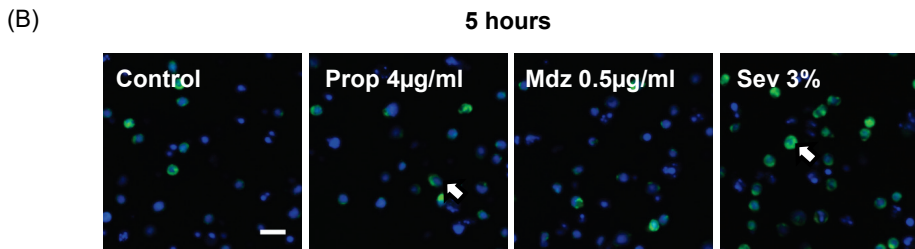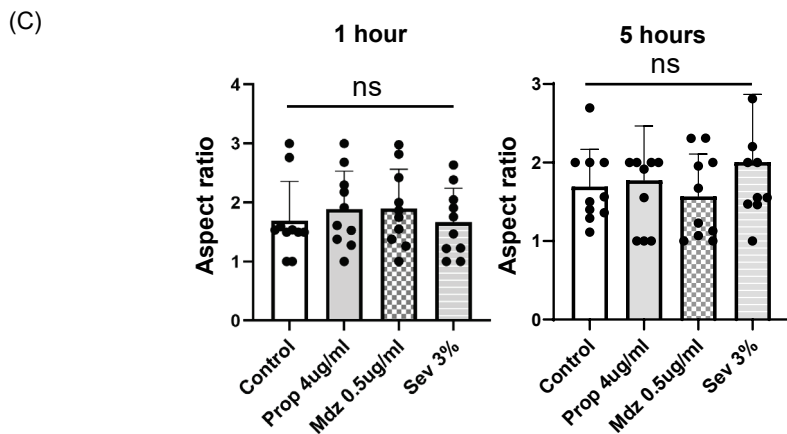

Supplement: Multimedia component 7 [file mmc7.pdf]
